# Anticholinergic Burden and Cognitive Performance in Patients With Schizophrenia: A Systematic Literature Review

**DOI:** 10.3389/fpsyt.2021.779607

**Published:** 2021-12-28

**Authors:** Rafaella Georgiou, Demetris Lamnisos, Konstantinos Giannakou

**Affiliations:** Department of Health Sciences, School of Sciences, European University Cyprus, Nicosia, Cyprus

**Keywords:** cognitive function, anticholinergic burden, schizophrenia, psychopharmacology, systematic review

## Abstract

**Objective:** Cognitive impairment in schizophrenia forms the key cause of the disease's disability, leading to serious functional, and socioeconomic implications. Dopaminergic-cholinergic balance is considered essential to cognitive performance in schizophrenia and patients are often treated with many drugs with anticholinergic properties. This study aims to examine the cognitive impact of anticholinergic burden in patients with schizophrenia.

**Methods:** A systematic literature review was performed on English-language studies published on PubMed, Embase, and Web of Science, from inception to June 2021, to identify research studies that examined the effect of anticholinergic load on cognition in clinically stable patients with schizophrenia. No restrictions on study design, age of participants, or geographical distribution were applied. Two researchers performed independently the screening and shortlisting of the eligible articles. A narrative synthesis of the main characteristics and findings of studies included was reported.

**Results:** In total, 17 articles of varying methodological design met the inclusion criteria. Three of them found statistically significant improvement in cognition after anticholinergic tapering without adverse effects. Thirteen studies found a statistically significant association between high anticholinergic burden and cognitive impairment (neurocognitive composite scores and individual cognitive domains such as learning and memory, executive function, processing speed), apart from a study, related to the specific characteristics of clozapine.

**Conclusions:** Medication with increased anticholinergic load has been found in most of the studies to negatively affect neurocognitive performance of patients with schizophrenia. However, the clinical and methodological heterogeneity of studies included limit our interpretation and conclusions.

## Introduction

Cholinergic neurotransmission plays a crucial role in both psychotic symptoms and cognitive disorders as well as treatment of schizophrenia (1). Specifically, an increased number of cholinergic neurons in the reticular formation of some patients with schizophrenia has been reported (2). Additionally, studies have found a decrease in muscarinic receptor levels in brain regions critical to the pathophysiology of schizophrenia (e.g., frontal cortex, basal ganglia, and hippocampus) (3–5) and particularly the alteration of M1 muscarinic receptors has been significantly associated with memory and learning deficits observed in the disease (6). Furthermore, studies of post-mortem brains from patients with schizophrenia, which exhibit a reduction of α7 nAChR expression in the hippocampus and cingulate cortex (7, 8) as well as genome-wide association studies that relate the risk for schizophrenia with the copy number variations of a locus containing the α7 nAChR, reveal the abnormal cholinergic regulation of the disease (9). The high smoking rates observed in schizophrenia may also be due to patients using nicotine, an nAChRs agonist, as a self-medication (10, 11). It is also worth noting that literature indicates a tight link between mesolimbic dopaminergic and basal forebrain cholinergic activity (12–14) and therefore this interaction may explain the integration of motivational functions with attentional functions. Abnormal mesolimbic dopaminergic activity is likely to alter cholinergic function and thus attentional performance, which is also supported by rodent models of attention impairment. According to Kozak's et al. findings (15), animals with sensitized mesolimbic dopaminergic functions exhibited cholinergic systems that remain “frozen” at baseline and unable to support attentional performance, while a different animal study showed that stimulation of mesolimbic–corticopetal cholinergic circuitry enhanced attention performance (16). Thus, increased understanding of cholinergic neurotransmission of cortical function can contribute particularly to the understanding of attentional dysfunction observed in schizophrenia.

In addition to findings that correlate the pathogenetic mechanism of the disease with abnormal cholinergic neurotransmission, patients with schizophrenia are often treated in clinical practice with anticholinergic drugs to control the extrapyramidal side effects that cause most antipsychotics, especially first generation (17–19). Furthermore, most of psychotropic medications used have, beyond the affinity of D2 dopamine receptors, also anticholinergic activity (19). Although antipsychotics may improve clinical symptoms of patients with schizophrenia, high doses or a combination of different types of antipsychotic drugs have been associated with a decrease in cognitive functions (20, 21). Tani et al. (22) argue that due to the disturbance of the cholinergic system in schizophrenia, any exogenous anticholinergic activity can cause endogenous anticholinergic activity and therefore the appearance and exacerbation of the symptoms of the disease. While there are many hypotheses about how antipsychotics are associated with the decline in cognitive abilities of patients with schizophrenia, there is evidence to suggest that the cumulative anticholinergic effect of the various pharmacotherapies may be an important factor. Therefore, one could assume that particularly cognitive domains such as attention, working memory, and spatial memory, which according to the literature are affected by cholinergic regulation may also be adversely affected by the accumulated anticholinergic load of pharmacotherapies.

Given that impaired cognitive performance is a strong determinant of the outcome of the disease as well one of the major causes of the disease's disability affecting the daily social, occupational functionality, autonomy, and independence (23, 24), there arises the need to investigate the association of medication anticholinergic load with the cognitive deficits in patients with schizophrenia. Thus, the aim of this systematic review is to summarize the findings of epidemiological studies that assessed the effect of anticholinergic burden on cognitive performance of patients with schizophrenia and to examine the importance of these findings in future research and clinical practice.

## Methods

For the conduction of this systematic review, we followed the Preferred Reporting Items for Systematic Reviews and Meta-Analyses guidelines (PRISMA) (25).

### Search Strategy

A systematic literature search in PubMed, Embase, and Web of Science was conducted by two independent authors (RG and KG) to identify articles that examined the effect of anticholinergic load on cognitive domains in patients with schizophrenia. The following terms were used: (anticholinergic OR antimuscarinic OR parasympatholytic) AND (cognitive OR brain OR memory OR dementia OR confusion) AND schizophrenia. All databases were searched from their inception through June 2021, restricted to English language publications. First, the title and abstract of each article was examined, and then the full texts of potentially eligible articles to be included in the systematic review were evaluated. A reference list of relevant studies was screened to identify additional studies.

### Eligibility Criteria

We used the Population, Intervention, Comparison, Outcomes and Study design (PICOS) approach (26) for the identification of included studies. Participants were only patients with schizophrenia regardless of age and gender, while for the intervention we considered the effect of anticholinergic load medication treatment. For the comparison, studies had a control/comparison group of patients or not were eligible for inclusion. The chosen outcome was the impact of anticholinergic load on cognitive domains in people with schizophrenia. For study design, any design was eligible for inclusion.

#### Intervention of Interest

Assessment of exposure's degree to drugs with anticholinergic properties is a combination of the daily dose, the affinity to muscarinic receptors, the permeability to the blood barrier, and drugs' general pharmacokinetic and pharmacodynamic characteristics. Therefore, many different scales and methods of classifying cumulative anticholinergic burden are available. Eligible intervention criteria were (a) the tapering of anticholinergic therapy or (b) the cumulative anticholinergic load ranked either by anticholinergic classification scales or calculated based on serum anticholinergic activity (SAA).

Anticholinergic Drug Scale (ADS): A classification system for rating anticholinergic potency of medicines based on the clinical experience, expert opinion, and *in vitro* pharmacological properties. Medicines with anticholinergic properties are ranked from 0 (none) to 3 (high) (27, 28). Anticholinergic cognitive burden (ACB) scale: A classification system, which ranks drugs based on serum anticholinergic activity or *in vitro* binding affinity with muscarinic receptors. Drugs are graded from 0 (no anticholinergicity) to 3 (high anticholinergicity with significant adverse cognitive effects) (29). Anticholinergic burden classification (ABC): A classification system which quantifies anticholinergic burden ranging from 0 (none) to 3 (high) based on serum anticholinergic activity and expert opinion (30). SAA: Serum levels of anticholinergic drugs measured with a competitive radioreceptor binding assay technique (e.g., by Tune and Coyle) (31).

#### Outcome of Interest

Since cognitive impairment in schizophrenia is observed in a variety of cognitive domains (e.g., attention, working memory, verbal memory, etc.) different diagnostic tools can be used for cognitive performance evaluation. The MATRICS Consensus Cognitive Battery (MCCB), although an FDA-approved neurocognition test battery, is usually only used to extract an overall score, whereas the assessment of complex domains such as executive function requires additional diagnostic tools (e.g., Wisconsin Card Sorting Test, Stroop Test, Trail Making Test) (32). Comprehensive neuropsychological assessments are often difficult in clinical practice to carry out, so there are several brief evaluation tools, which can provide useful information on the outcome of interest (e.g., Brief Assessment of Cognition for Schizophrenia; BACS) (33). Therefore, due to the complexity of the cognitive outcome, different neurocognitive assessment batteries meet the inclusion criteria. However, studies with functional and structural imaging outcomes are beyond the scope of this article and are excluded from this systematic review because they lead to greater variability and lack of outcome coherence.

### Data Extraction and Quality Assessment

Two authors (RG & KG) extracted data independently and any discrepancy was resolved by discussion with the third author (DL). A standardized data extraction form was developed for our systematic review and it was used to minimize data entry errors and improve validity and reliability. The extracted data included first author, publication year, country of origin, design of the study, sample size, characteristics of the study population, study groups, considered confounding variables, and summary of findings.

The methodological quality of cohort and case-control studies was performed by the Newcastle-Ottawa Scale (NOS) (34). The NOS is a validated scale to assess non-randomized control trials and each study can be awarded up to nine stars. Each study is assessed on eight questions, in three groups: quality of selection, comparability between the groups and outcome. Studies with NOS values ≥6 were considered moderate to high-quality studies (34). Studies with NOS values lower than 6 were considered low-quality studies. The Newcastle-Ottawa quality assessment scale has been adapted for cross-sectional studies and was used for the methodological quality of the included cross-sectional studies (35). Quality assessment of clinical trial studies was carried out using the validated modified Jadad scale (36, 37). Modified Jadad scale is a scoring system that allows a maximum score of 8, assessing randomization, blinding, presentation of withdrawals/dropouts, inclusion/exclusion criteria, adverse effects, and statistical analysis. Clinical studies with a score ranging from 4 to 8 are considered moderate to high methodological quality design, whereas lower than 4 are considered low methodological design.

## Results

### Study Selection and Characteristics

**Figure 1:**
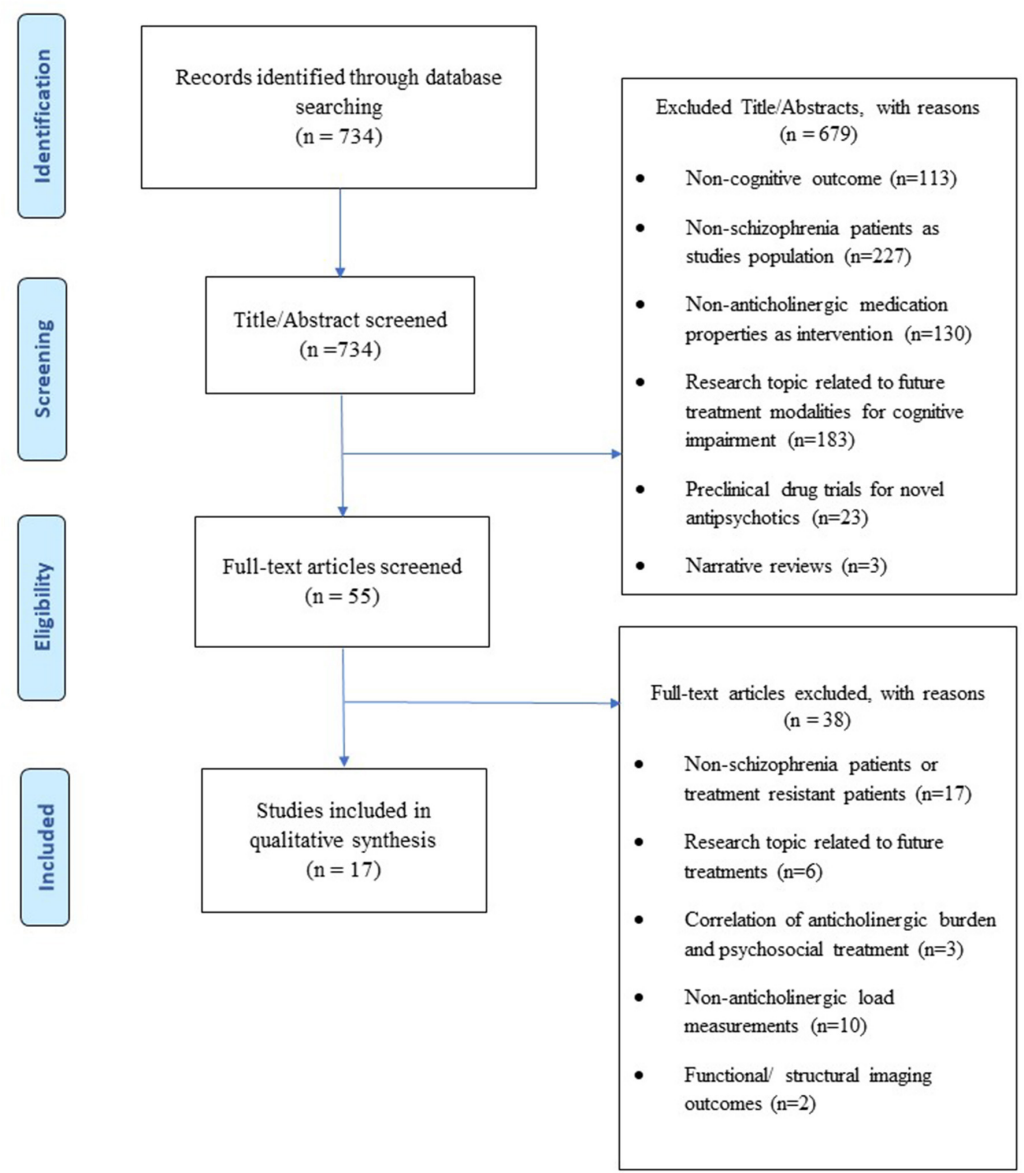
PRISMA flow diagram of search strategy and final selection of articles.

From the initial electronic search, we found a total of 734 articles, 679 of which were excluded based on title and abstract review. The full text of the remaining 55 was checked for eligibility and 38 of them were excluded. Overall, a total of 17 articles were included in this systematic review (Figure 1). No further articles were identified through searching the reference list of reviews identified during the initial search nor the reference list of the included articles. Meta-analysis was not possible to be conducted due to the heterogeneity of the data and the clinical and methodological variability of the studies included. Therefore, a narrative synthesis of the main characteristics and findings of the studies included was reported.

The publication years were from 1982 to 2021. The countries covered are the United States, China, South Korea, Germany, Canada, Japan, and Israel. The number of participants included in the studies ranged from 15 to 1,120. Most of the studies included, recruited clinically stable adults in inpatient setting, with no history of pre-existing organic impairment, unstable medical or neurological conditions, or prohibited drug abuse. As regards to the study design, the majority were cross-sectional studies (38–47). Additionally, one retrospective (19), four prospective cohort studies (48–51), and two randomized clinical trials (52, 53) were included. Regarding the intervention assessment method, three studies assessed cognitive performance after anticholinergic tapering, six studies evaluated the influence of serum anticholinergic activity (SAA) on cognition, and eight studies assessed the influence of medication anticholinergic burden using anticholinergic classification scales on cognitive function.

Tables 1–3 summarize the demographics of the participants, the main characteristics, and findings of the studies included accordingly to the intervention assessment method. Below are presented in detail the main results of the studies that refer to the impact of medication anticholinergic burden in specific cognitive domains evaluated with different neurocognitive tasks in patients with schizophrenia.

**Table 1:** Qualitative analysis with the main characteristics of studies related to the influence of anticholinergic tapering on cognition in schizophrenia.

**References, study design**	**Country**	**Setting**	**Population**	**Age (years)**	**% Female**	**Study groups**	**Cofounders studied**	**Intervention assessment method**	**Outcome assessment method**	**Summary of findings**
Drimer et al. (49), prospective cohort study	Israel	Inpatients from Abarbanel Mental Health Center, Bat-Yam, Israel	*n* = 27	Mean age 65.7 years	51.85% female	No control group	N/A	Anticholinergic tapering	ADAS–Cog	Biperiden tapering showed significant improvement in ideational praxis, orientation, and overall score of ADAS–Cog. Improvement correlated with previous dose of biperiden. No adverse events/extrapyramidal symptoms
Desmarais et al. (48), prospective cohort study	Canada	Outpatients from Schizophrenia Tertiary Services outpatient clinic of the McGill University Health Center	*n* = 20 with schizophrenia or schizoaffective disorder	52.7 ± 7.8 years	42.86% female	No control group	Gender, Age, Education level, Age at onset of illness, Length of illness, Presence of parkinsonism, dystonia, or tardive dyskinesia	Anticholinergic tapering	ESRS, BACS or BECS (French version) depending on patients' language, PANSS, CGI-S CGI-I	Significant improvement on BACS z score at weeks 6, 8, and 12, especially on motor and symbol-coding tasks after anticholinergic tapering. No significant effects on the PANSS, CGI-S, and CGI-I
Ogino et al. (50), prospective cohort study	Japan	Inpatients and Outpatients from St. Marianna University School of Medicine Hospital and Ofuji Hospital	*n* = 24 biperiden tapering group schizophrenia patients, *n* = 10 control group	Biperiden tapering group schizophrenia patients: 35.7 ± 12 years Control group: 43.5 ± 8.7 years	Biperiden tapering group schizophrenia patients: 45.83% female Control group: 50% female	Biperiden tapering group, control group schizophrenia patients	Gender, age, education, age at onset of illness, duration of untreated psychosis, duration of biperiden administration/or mean daily dose of biperiden, antipsychotic drugs, or benzodiazepines	Biperiden tapering	BACS-J, SQLS-J, PANSS	Significant improvements in attention, processing speed, and composite score (BACS-J), in psychosocial condition score (SQLS-J) and general psychopathology score (PANSS) after biperiden tapering

*ADAS-cog, Alzheimer's disease assessment scale-cognitive; BACS, Brief assessment of cognition in schizophrenia; BACS-J, Brief assessment of cognition in schizophrenia-Japan, BECS, brève evaluation de la cognition en schizophrénie; CGI-I, Clinical global impression—improvement scales; CGI-S, Clinical global impression-severity scales; ESRS, Extrapyramidal symptom rating scale; PANSS, Positive and negative syndrome scale; SQLS-J, Schizophrenia quality of life scale-Japan*.

**Table 2:** Qualitative analysis with the main characteristics of studies related to the influence of medication anticholinergic burden on cognition in schizophrenia.

**References, study design**	**Country**	**Setting**	**Population**	**Age (years)**	**% Female**	**Study groups**	**Cofounders studied**	**Intervention assessment method**	**Outcome assessment method**	**Summary of findings**
Kim et al. (40), cross sectional study	South Korea	Inpatients from a university hospital and 2 mental hospitals	*n* = 60, Low ADS *n* = 31 High ADS *n* = 29	Low ADS: 35.61 ± 7.26 years High ADS: 42.59 ± 10.66 years	Low ADS: 25.81% female, High ADS: 31.03% female	ADS ≥ 3 group and ADS < 3	Gender, age, depression, education	ADS	MCCB for cognitive functions/UPSA for daily living functions	Statistically negative association between high anticholinergic burden and poorer cognitive (composite MCCB score) and daily living functions (total UPSA score)
Ang et al. (38), cross sectional study	China	Outpatients/ inpatients from the Institute of Mental Health, Singapore, community care centers and rehabilitation centers in Singapore	*n* = 705	39.18 ± 9.71 years	47.2% female	No control group	Duration and severity of illness, antipsychotic dose, smoking status, age, gender	ABS and ADS	Neuropsychological battery (JLO, WASI–II, CPT-IP, BACS)	Anticholinergic burden was negatively correlated with cognitive performance in global cognition (executive function, memory/fluidity, processing speed) but due to the small size of the association, the clinical significance is doubtful
Eum et al. (39), cross sectional study	United States	From the bipolar-schizophrenia network on intermediate phenotypes (B-SNIP) consortium	*n* = 206 with schizophrenia *n* = 131 with schizoaffective disorder *n* = 146 with psychotic bipolar disorder	With schizophrenia: 36.37 ± 13.21 years, with schizoaffective disorder: 36.83 ± 11.84 years, with psychotic bipolar disorder: 35.08 ± 12.20 years	With schizophrenia: 33.5% female, with schizoaffective disorder: 59.5% female, with psychotic bipolar disorder: 62.3% female	No control group	Age, gender, symptom severity (PANSS total score), antipsychotic burden (CPZeq), education, race	ADS	BACS	ADS scores ≥ 4 had lower composite BACS scores than those with ADS < 4 (p=0.004). Verbal memory showed statistically worse performance in the high anticholinergic load group
Rehse et al. (19), retrospective cohort study	Germany	Psychiatric outpatient unit for cognitive training of the psychiatric department at the Heidelberg University Hospital, Germany	*n* = 104 with schizophrenia	28.2 ± 8.6 years	59.5% female	Group A–ADD receivers, Group B–ADD + CDD receivers	Age, gender, education level, time since onset of illness	ADD was converted into RIS-Eq/CPZ-Eq CDD was converted into BZT-Eq	MCCB	Significant negatively correlation of ADD and tasks of information processing speed and verbal memory. No statistically significant correlation of CDD and cognitive performance
Minzenberg et al. (41), cross sectional study	United States	Outpatients from San Francisco VA Medical Center and surrounding community	*n* = 106 with schizophrenia or schizoaffective disorder *n* = 50 control group	Patients:39.9 ± 11.3 years Control group: 39.4 ± 12.6	Patients: 4% female Control group: 42% female	patients, healthy subjects	Age, Parental education, Parental occupation level, symptoms severity, global function	Pharmacological index from published studies Clinical index from clinician ratings of anticholinergic medication adverse effects	Neuropsychological battery (WAIS-R, TMT-A/B, digit span/visual span from WMS-R, wisconsin card sorting test, stroop color and word test, victoria version, rey-osterrieth complex figure design, california verbal learning test, facial memory from test of memory and learning, serial visuospatial learning test, controlled oral word association test, ruff figural fluency test, finger tapping test) extended version of PANSS	Anticholinergic load associated with lower scores on attention, declarative memory, and verbal memory
Tsoutsoulas et al. (45), cross sectional study	Canada	Community-Dwelling patients	*n* = 60	≥50 years old		No control group	N/A	ACB	CANTAB Alzheimer's Dementia Battery for cognitive deficits and Repeatable Battery for neuropsycological status measures	Anticholinergic burden had significant negative impact in spatial working, short-term memory, visuospatial ability, and a negative trend level of correlation with learning performance. No adverse effects on attention, executive function, language, or reaction time
Joshi et al. (47), cross sectional study	United States	Outpatients from five U.S. universities -part of the Consortium on the Genetics of Schizophrenia−2	*n* = 1,120	Mean age: 46 years old	Patients: 30% female	ACB = 0, ACB = 1 or 2 (low), ACB = 3 or 4 (moderate), ACB = 5 or 6 (high), ACB > 6 (very high) groups	Age, Demographic characteristic, illeness severity, Antipsychotic burden (CPZeq), clinical symptoms	ACB	Penn Computerized Neurocognitive Battery (PCNB)	Anticholinergic burden had significant negative impact on cognitive performance across all cognitive domains
Sweeney et al. (43), cross sectional study	United States	Inpatients	*n* = 44 with schizophrenia or schizoaffective disorder	28.5 ± 8.6 years range from 18 to 54 years	40.91% female	No control group	Clinical status, IQ, other psychotropic medication	Pharmacological history data of antipsychotic (CPZ) equivalent dosages) and anticholinergic (benzotropine) medication treatment	Neuropsychological tests battery for motor speed (finger tapping test), psychomotor skills (TMT, WAIS-R, Digit Symbol), visual spatial skills (WAIS-R Block Design, Benton's JLO), verbal fluency (Benton's COWA), verbal memory (Rey AVLT), flexibility of cognitive set (WCST)	Higher antipsychotic doses associated with worse performance on psychomotor speed, attention, and Wisconsin Card Sort. Higher anticholinergic dose was associated with worse performance on verbal learning, verbal fluency, and motor speed.

*AA, Anticholinergic activity; ADD, Antipsychotic daily dose; ADS, Anticholinergic drug scale; ANT, Attention network test; AVLT, Auditory verbal learning test; BACS, Brief assessment of cognition in schizophrenia; BZT-Eq, Benztropine equivalent doses; CANTAB, Cambridge neuropsychological test automated battery; CDD, Cholinergic daily dose; COWA, Controlled oral word association test; CPT-IP, Continuous performance test–identical Pairs version; CPZeq, Chlorpromazine equivalents; DLPFC, dorsolateral prefrontal cortex; JLO, Judgment of line orientation test; MATRICS, Measurement and treatment research to improve cognition in schizophrenia; MCCB, MATRICS consensus cognitive battery; N/A, Not available; PANSS, Positive and negative syndrome scale; RCT, Randomized clinical trial; RIS-Eq, risperidone equivalent doses; RWT, Regensburger wortfluessigkeits-test; SAA, Serum anticholinergic activity; TMT-A/B, Trail making test A and B; WAIS-R, Wechsler adult intelligence scale-revised; WASI–II, Wechsler abbreviated scale of intelligence matrix reasoning; WCST, Wisconsin card sorting test; WMS-R, Wechsler memory scales—revised*.

**Table 3:** Qualitative analysis with the main characteristics of studies related to the influence of SAA on cognition in schizophrenia.

**References, study design**	**Country**	**Setting**	**Population**	**Age (years)**	**% Female**	**Study groups**	**Cofounders studied**	**Intervention assessment method**	**Outcome assessment method**	**Summary of findings**
Vinogradov et al. (53), Single blind randomized clinical trial	United States	Outpatients from community mental health centers	*n* = 55, auditory training group *n* = 25; control group *n* = 24	Auditory training group: 41.44 ± 11,06 years Control Group: 46.38 ± 8.97 years	Auditory training group: 32% female Control group: 28% female	Auditory training group and control group	Age, gender, education, and symptom severity, IQ	SAA with radioreceptor assay	Neurocognitive battery based on MATRICS	Higher SAA significantly correlated with worse verbal working memory, verbal learning-memory, and global cognition change after auditory training
Tune et al. (46), cross sectional study	United States	Outpatients	*n* = 24 from 20 to 58 years	Mean age 35.7	45.83% female	No control group	N/A	SAA with radioreceptor assay of Creese and Snyder for neuroleptics and radioreceptor assay of Tune and Coyle for anticholinergics	Free recall memory test, WAIS, structured interview by psychiatrist	Significant correlation between high SAA and recall test performance
Tracy et al. (44), cross sectional study	United States	Patients at Norristown State Hospital	*n* = 38	39.7 ± 10.2 years	32% female	High/low anticholinergic group	Gender, age, education level, Smoking, alcohol duration of illness, other neurological diseases, antipsychotic dose	SAA with radioreceptor assay of Tune and Coyle	CPT-IP for selective attention, Stroop Test for “inhibitory” executive control, Digit Vigilance Test for sustained attention, a single verbal memory task for automatic and effortful memory, finger tapping test for psychomotor speed	Significant correlation between high SAA and worse performance on executive control and effortful memory
Perlick et al. (42), cross sectional study	United States	Inpatients from psychiatric hospital in New York	*n* = 17	Mean age 33.4 years	29.41% female	No control group	IQ, Age, organic impairment related to mental capacity, serum neuroleptic load	SAA with radioreceptor assay of Tune and Coyle (31) for anticholinergics and Tune et al. (46) for neuroleptics	Neuropsychological tests battery (WAIS-R, Benton's revised visual retention test, mattis-kovner memory inventory) brief psychiatric rating scale	Significant correlation between high SAA and verbal recall memory. No association between serum anticholinergicity and recognition memory
Hitri et al. (52), randomized clinical trial	United States	Inpatients from Augusta Veterans Administration Hospital, Georgia	*n* = 15	Range from 28 to 60 years	0% female	Group benztropine; *n* = 5, Group trihexyphenidyl; *n* = 5, Group amantadine; *n* = 5	N/A	SAA with radioreceptor assay of Tune and Coyle for anticholinergics and Creese and Snyder for neuroleptics	Neuropsychological tests battery for attention, concentration, memory (digit span, selective reminding memory task by Buschke)	Higher SAA correlated with reduction of short-term recall performance but not with long-term memory function
Tracy et al. (51), prospective cohort study	United States	Inpatients	*n* = 22	44.7 ± 8.4 years	45.5% female	Group clozapine; *n* = 15, Group risperidone; *n* = 7	Age, Age at onset of illness, other neurological disorders, medication possible to affect cognition, race, gender, education	SAA with radioreceptor assay of Tune and Coyle for anticholinergics	Neurocognitive test battery	Higher SAA of patients treated with clozapine than risperidone but no differences on cognitive functions

*AA, Anticholinergic activity; ADD, Antipsychotic daily dose; ADS, Anticholinergic drug scale; ANT, Attention network test; AVLT, Auditory verbal learning test; BACS, Brief assessment of cognition in schizophrenia; BZT-Eq, Benztropine equivalent doses; CANTAB, Cambridge neuropsychological test automated battery; CDD, Cholinergic daily dose; COWA, Controlled oral word association test; CPT-IP, Continuous performance test–identical Pairs version; CPZeq, Chlorpromazine equivalents; DLPFC, dorsolateral prefrontal cortex; JLO, Judgment of line orientation test; MATRICS, Measurement and treatment research to improve cognition in schizophrenia; MCCB, MATRICS consensus cognitive battery; N/A, Not available; PANSS, Positive and negative syndrome scale; RCT, Randomized clinical trial; RIS-Eq, risperidone equivalent doses; RWT, Regensburger wortfluessigkeits-test; SAA, Serum anticholinergic activity; TMT-A/B, Trail making test A and B; WAIS-R, Wechsler adult intelligence scale-revised; WASI–II, Wechsler abbreviated scale of intelligence matrix reasoning; WCST, Wisconsin card sorting test; WMS-R, Wechsler memory scales—revised*.

### Anticholinergic Tapering and Cognitive Performance

The characteristics of studies included that examine the association of anticholinergic tapering with neurocognitive performance are presented in Table 1. In light of the administration of anticholinergic agents to control extrapyramidal symptoms, three prospective studies (48–50) examined the effect of anticholinergic tapering on cognitive functions. Despite the small number of participants (*n* = 27, *n* = 34, and *n* = 20), after successful discontinuation of anticholinergic treatment by most participants, no adverse effects to psychopathology or extrapyramidal symptoms were found, while a significant improvement in the composite/overall scores of the neurocognitive batteries used (BACS, BACS-J, ADAS–Cog) during the follow-up weeks was observed. Anticholinergic tapering showed improvement over baseline in cognitive tasks that mainly assess the domains of processing speed, attention, ideational Praxis, and Orientation.

In the Desmarais et al. (48) patients with schizophrenia or schizoaffective disorder were recruited and treated with a long-term stable dose of antipsychotics and anticholinergics (7.3 ± 3.3 mg/day), whereas the Ogino et al. (50) included patients with paranoid type of schizophrenia who received stable dose of second-generation antipsychotics with co-administered biperiden (2.2 ± 0.8 mg/day) as well as a control group, which neutralized the confounding factor of the practice effect. In the Desmarais et al. study (48) anticholinergic tapering revealed significant improvement in the composite z score of BACS at weeks 6, 8, and 12 compared to the starting point (*p* = 0.029, *p* = 0.001, and *p* = 0.002, respectively), with average corresponding differences −0.287 (95% CI: −0.023 to −0.552), −0.416 (95% CI: −0.146 to −0.686), and −0.517 (95% CI: −0.163 to −0.871), whereas in the Ogino et al. (50) in the overall interaction (time × groups), statistical significance appeared in the composite score assessed with BACS-J [*F*_(1,32)_ = 6.06, *p* = 0.02], 4 weeks after discontinuation. In both studies improvement was observed in tasks that incorporate the domains of attention and processing speed. Particularly in the Desmarais et al. (48) significant improvements were observed mainly in motor tasks, in which in the 12th week the average z value was significantly higher than the baseline by an average difference of −0.476 (p = 0.023) with 95% CI from −0.049 to −0.902, as well as in symbol coding tasks (*p* = 0.043) with an average improvement of −0.279 and 95% CI from −0.006 to −0.552. As regards the Ogino et al. (50), in the analysis between biperiden discontinuation group and control group, significant improvements were observed in symbol coding tasks in the anticholinergic discontinuation group [*F*_(1,32)_ = 4.75, *p* = 0.04] and regarding the analysis among the participants of the biperiden discontinuation group, significant time changes in verbal fluency [*F*_(1,32)_ = 6.56, *p* = 0.02] as well as in symbol coding task [*F*_(1,32)_ = 6.21, *p* = 0.02] were observed. In the overall interaction (time × groups), also a statistical significance appeared in the symbol coding test [*F*_(1,32)_ = 10.66, *p* = 0.003]. Drimer et al. (49) used a different diagnostic tool, ADAS-Cog (Alzheimer's Disease Assessment Scale-Cognitive), with elderly patients above the age of 60 receiving biperiden (2–6 mg/day) for at least 1 year. Statistically significant improvements related to previous biperiden doses were also found. Specifically, improvements were presented in the ideational Praxis subscales (*t* = 2.63, *p* < 0.02), orientation (*t* = 2.41, *p* < 0.03), and overall score (*t* = 2.43, *p* < 0.03) 10 days after anticholinergic discontinuation.

### Anticholinergic Burden and Cognitive Performance

#### Anticholinergic Burden and Composite Cognitive Scores/Global Cognition

Out of the six studies that evaluated the estimated overall cognitive performance with the combination of representative cognitive tests from multiple domains, five provided evidence that increased medication anticholinergic burden was significantly associated with decreased global cognition or composite scores comprised of several different cognitive tests.

Specifically, in the study of Kim et al. (40), in which participants were classified into a “low ADS” and a “high ADS” group with a score of < or ≥3 respectively, high anticholinergic load of medication (ADS ≥ 3) was associated with worse cognitive performance, assessed with MCCB (composite MCCB score, *r* = −0.512, *p* < 0.001). The correlation was also confirmed through regression analysis (composite MCCB score, R^2^ = 0.262, *p* < 0.001). Although the explanatory power decreased after adjusted analysis, anticholinergic load continued to have a statistically negative impact on the MCCB composite score (*p* = 0.013). The recent study of Joshi et al. with a large sample size (47) was also in agreement with these findings using a different intervention and outcome assessment method. Specifically, patients with higher scores based on Anticholinergic Cognitive Burden (ACB) scale demonstrated worse cognitive performance across all cognitive domains assessed with Penn Computerized Neurocognitive Battery (PCNB) compared to patients with low ACB scores. The cross-sectional study of Eum et al. (39) detected a different threshold (ADS ≥ 4) of the adverse effects of anticholinergic burden on clinically stable patients with schizophrenia. Subjects with an ADS score ≥4 demonstrated statistically significant poorer cognitive composite scores (*p* = 0.004) evaluated with the BACS, by approximately a mean of 0.58 lower SD, than patients with an ADS score <4. In addition, an also large study in terms of the number of participants (*n* = 705) found that the anticholinergic burden had a significant negative correlation with cognitive performance in global cognition however the effect size was small for anticholinergic burden scale (ABC), (Cohen f^2^ = 0.008) and for anticholinergic drug scale (ADS), (Cohen f^2^ = 0.017), and therefore the clinical significance of the result is not apparent (38). The different study approach of the randomized controlled clinical trial of Vinogradov et al. (53) also provided evidence that increased anticholinergic load negatively affects the response to cognitive training with the intensive auditory training “based on neuroplasticity.” Increased SAA which showed a negative correlation with the improvement of global cognition in participants in audio training (Pearson's *r* = −0.46, *p* < 0.02) represented 20% of the variation in global cognition change, regardless of age, IQ, or severity of symptoms. On the other hand, the study of Tracy et al. (51) including 22 chronic patients with schizophrenia found no effect of the anticholinergic load on the cognitive measure. Particularly, differences in global cognition were not observed between patients with significantly higher serum anticholinergic levels (*p* < 0.001) treated with antipsychotic clozapine compared to those treated with antipsychotic risperidone (lower SAA).

#### Anticholinergic Burden and Learning, Memory, and Verbal Skills

In total, six studies with different methodological design and a variety of medication anticholinergicity measures reported significant association between increased anticholinergic burden and decline in several types of memory (declarative, verbal, short-term) as well as the domains of learning and language/verbal skills.

Minzenberg et al. (41) using clinical and pharmacological *in vitro* indicators reported a significant association of high anticholinergic load with worse declarative memory performance. Specifically, the study found that an increase in anticholinergic load from 0.5 to 4 mg/day equivalent pharmacological benztropine can lead to a reduction of up to 1.7 SD of the California verbal learning test performance. In addition, Sweeney et al. (43) found that higher anticholinergic doses were also correlated with worse performance on verbal fluency (Verbal Fluency test) and verbal memory tasks (Rey AVLT). Similarly, memory/fluency performance although the small effect size was also reported to have a significant negative association with anticholinergic load evaluated with ABC and ADS scales in Ang's et al. (38). Furthermore, Eum's et al. cross-sectional study (39) reported statistically worse performance in the high anticholinergic load group (ADS ≥ 4) compared to the low anticholinergic group ADS < 4 in verbal memory task with a difference of 0.55 SDs. Significant are also the findings of the Vinogradov et al. study (53) in which high SAA was significantly associated with worse performance in the MCCB domain of verbal learning and memory (*r* = −0.29, *p* < 0.04), with which after regression analysis, SAA showed a common variation of 7%, after controlling for the impact of age, IQ, and severity of symptoms. In addition, the cognitive effects of anticholinergic load, evaluated according to a different anticholinergic scale (ACB scale), in older patients with schizophrenia (≥50 years), bear a similarity to cognitive dysfunction that occur in the early stages of Alzheimer's disease. High anticholinergic load was correlated with short-term memory decline (*p* = 0.004) evaluated with Alzheimer's Dementia Battery and the Repeatable Battery for the Assessment of Neuropsychological Status. It is also worth noting the emergence of a correlation tendency between high anticholinergic load and poorer learning performance (45).

##### Anticholinergic Burden and Retrieval

As regards specifically the performance of memory recall or retrieval (free recall, cued recall, and recognition), which refers to the mental process of retrieval of the information/events that were previously encoded and stored in the brain, in total four studies found a significant negative association with high SAA.

Specifically, in Tune's et al. cross-sectional study (46) the performance of the free recall test was of statistical importance (*r* = 0.51, *p* < 0.01) demonstrating a negative effect of the high anticholinergic load on recent or working memory. On the other hand, a cross sectional study (42) with also a small sample size (*n* = 17) of chronic patients, similar age range but serum anticholinergicity due more to neuroleptic treatment than to antiparkinsonian drugs, was only partly in line with Tune's et al. (46) on the correlation between anticholinergic load and recall memory impairment (*r* = −0.54, *p* = 0.01). As regards the recognition memory, which was evaluated with the Mattis-Kovner inventory test, no association with the anticholinergic load (*r* = 0.28, *p* > 0.10) was found. Recall performance also declined (List Recall=total free recall; *t* = −2.16, *p* = 0.037) in the group with the high serum anticholinergic load in the study of Tracy et al. (44) in which after statistical analysis, this task showed common variance with serum anticholinergicity of 16% (*r* = 0.40). In addition, the Hitri et al. (52) found that during the period of anticholinergic treatment, in which there was an increase in SAA, there was a decrease in the number of recall words by 22% compared to the starting point. Short-term recall memory decreased by 31%, but long-term memory did not show any statistically significant change due to the anticholinergic agents administered.

#### Anticholinergic Burden and Executive Functions

Executive function performance, which refers to a set of high-order cognitive processes such as working memory, inhibitory control, cognitive flexibility, planning, reasoning, and problem solving, was reported by five studies to be negatively affected with the increase of medication anticholinergic burden.

As regards working memory that is generally considered a key executive function domain, Vinogradov et al. (53) found that high SAA was significantly associated with worse performance in the MCCB domain of verbal working memory (*r* = −0.41, *p* < 0.04). Furthermore, high anticholinergic load assessed with ACB was also correlated with worse performance in spatial working memory (*p* = 0.04) in the study of Tsoutsoulas et al. (45).

In addition, in the Tracy et al. (44) patients treated only with a stable dose of single antipsychotic medication, higher SAA was associated with worse performance in Executive Control tests (Stroop interference index; *t* = −2,6, *p* = 0.015), in which after statistical analysis, the task showed common variance with serum anticholinergicity of 19% (*r* = −0.45). Similarly, in the Ang et al. (38) executive function performance (Judgment of Line Orientation test and Matrix Reasoning test) was also of significant importance; however, the effect size of both ABS and ADS scales was small questioning its clinical significance. It is also worth noting that a trend of effect on executive function was also observed in the retrospective cohort study of Rehse et al. (19) with the increase in daily pharmacological doses of anticholinergic drugs (CDD) (*b* = 0.280, *p* < 0.10) but is likely to be symptomatic as no significant results were observed under the increasing CDD load neither in the whole sample nor in the subgroups.

#### Anticholinergic Burden and Other Cognitive Domains

Furthermore, the domains of processing speed (including non-motor/cognitive activity or motor/physical activity), divided attention, and visual-spatial ability have been reported to be negatively associated with high medication anticholinergic burden.

Specifically, higher doses of anticholinergic benztropine were correlated with worse performance on motor speed (Finger Tapping, Digit Symbol) in Sweeney et al. (43) with clinically stable patients with schizophrenia, aged from 18 to 54 years old. On the other hand, in the study of Rehse et al. (19), CDD did not show significant influence in any cognitive field in the regression analysis of the entire sample; however, in the subgroup examination regarding antipsychotics' low or high anticholinergic binding profile, the increase in the anticholinergic load was associated with significantly lower data processing speed (*b* = 0.292, *p* < 0.05), as opposed to treatment with antipsychotics without anticholinergic properties. With the application of locally weighted scatterplot smoothing tool to verify the relationship of cognitive function with the increase in anticholinergic daily dose, although the tendency for reduced speed of information processing was confirmed by increasing CDD, a paradox was observed, since the group of the highest anticholinergic dose (BZT-Eq > 20) exhibited a higher rate of information processing than the group of 0 < BZT-Eq < 15. Nevertheless, this could be attributed either to insufficient data on anticholinergic equivalent doses for certain drugs or to the specific pharmacological properties of clozapine taken by all patients as the major antipsychotic drug, which could compensate for the adverse anticholinergic effects. Poorer processing speed/vigilance performance was also associated with the high anticholinergic burden in the study of Ang et al. (38), however the effect size was small for both anticholinergic classification scales (ABS and ADS).

Furthermore, the domain of divided attention was reported by Minzenberg et al. (41) to be negatively correlated with high anticholinergic load evaluated with pharmacological index from published studies and clinical index from clinician ratings of anticholinergic medication adverse effects (45). In addition, high ACB scores were correlated with worse visuospatial ability performance (*p* = 0.02) evaluated with Alzheimer's Dementia Battery and the Repeatable Battery for the Assessment of Neuropsychological Status in Tsoutsoulas et al. (45).

### Quality Assessment

Table 4 presents the results of the quality assessment based on the Newcastle-Ottawa scale for cohort studies. The range of methodological quality score was between 3 and 9. Four articles (19, 48, 50, 51) had a medium quality to high quality and the one (49) had a low methodological quality. Based on the Newcastle-Ottawa quality assessment scale adapted for cross sectional studies, the range of methodological quality score of the included studies was between 6 and 9 (Table 5). Lastly, our quality assessment for clinical trials, presented in Table 6, shows that two studies (52, 53) range in a score of between 3 and 3.5, indicating low methodological quality.

**Table 4:** Quality assessment of cohort studies (34).

**References**	**Selection**				**Comparability**	**Outcome**			**Total quality score**
	**(1)** **Representativeness of the exposed cohort**	**(2)** **Selection of the non-exposed cohort**	**(3)** **Ascertainment of exposure**	**(4)** **Demonstration that outcome of interest was not present at start of study**	**(1)** **Comparability of cohorts on the basis of the design or analysis^**a**^**	**(1)** **Assessment of outcome**	**(2)** **Was follow-up long enough for outcomes to occur?**	**(3) Adequacy of follow up of cohorts**	
Rehse et al. (19)	⋆	⋆	⋆		⋆⋆	⋆	⋆	⋆	8
Desmarais et al. (48)	⋆		⋆	⋆		⋆	⋆	⋆	6
Ogino et al. (50)	⋆	⋆	⋆	⋆	⋆⋆	⋆	⋆	⋆	9
Drimer et al. (49)			⋆				⋆	⋆	3
Tracy et al. (51)	⋆	⋆	⋆	⋆	⋆⋆	⋆		⋆	8

a *A maximum of 2 stars can be awarded for this item. A study controlling for age receives one star, and a study controlling for other major risk factors receives an additional star*.

**Table 5:** Quality assessment of cross-sectional studies (35).

**References**	**Selection** ^**a**^				**Comparability^**b**^**	**Outcome** ^**c**^		**Total quality score**
	**(1)** **Representativeness of the sample**	**(2)** **Sample size**	**(3)** **Non-responders**	**(4)** **Ascertainment of the exposure**	**(1)** **Comparability of subjects on the basis of the design or analysis**	**(1)** **Assessment of the outcome**	**(2)** **Statistical test**	
Joshi et al. (47)	⋆	⋆		⋆	⋆⋆	⋆⋆	⋆	8
Kim et al. (40)	⋆			⋆⋆	⋆⋆	⋆⋆	⋆	8
Ang et al. (38)	⋆	⋆		⋆⋆	⋆⋆	⋆⋆	⋆	9
Eum et al. (39)	⋆	⋆		⋆⋆	⋆⋆	⋆⋆	⋆	9
Minzenberg et al. (41)	⋆	⋆		⋆	⋆	⋆⋆	⋆	7
Tune et al. (46)	⋆			⋆⋆	⋆	⋆⋆		6
Tracy et al. (44)	⋆			⋆⋆	⋆⋆	⋆⋆	⋆	8
Perlick et al. (42)	⋆			⋆⋆	⋆⋆	⋆⋆	⋆	8
Tsoutsoulas et al. (45)		⋆		⋆⋆	⋆	⋆⋆	⋆	7
Sweeney et al. (43)	⋆			⋆	⋆	⋆⋆	⋆	6

a *A maximum of 5 stars can be awarded for the selection*.

b *A maximum of 2 stars can be awarded for the comparability*.

c *A maximum of 3 stars can be awarded for the outcome*.

**Table 6:** Quality assessment of clinical trials (36, 37).

**Corresponding author**	**Was the research described as randomize?**	**Was the approach of randomization appropriate?**	**Was the research described as blinding?^**a**^**	**Was the approach of blinding appropriate?**	**Was there a presentation of withdrawals and dropouts?**	**Was there a presentation of the inclusion/ exclusion criteria?**	**Was the approach used to assess adverse effects described?**	**Was the approach of statistical analysis described?**	**Total score**
Vinogradov et al. (53)	+1	0	0.5	0	+1	0	0	+1	3.5
Hitri et al. (52)	+1	0	0	0	0	0	+1	+1	3

a *Single-blind get 0.5 score and Double-blind get +1 score*.

## Discussion

To our knowledge, this is the first systematic review to examine the effect of cumulative anticholinergic burden on neurocognitive performance in patients with schizophrenia. Summing up the results of the studies included, it is largely concluded that medication with an increased anticholinergic load was observed in most studies as likely to affect the cognitive and daily living functions of patients with schizophrenia. Notably, the affected cognitive domains despite the abnormal cholinergic neurotransmission observed in schizophrenia are in line with our knowledge of the effect of cholinergic modulation on cognitive processes in healthy individuals (54). In the majority of studies, the increase in anticholinergic load was associated with impaired global cognition or decreased composite scores of several neurocognitive batteries as well as with decline of individual cognitive domains such as learning and memory, processing speed, executive function, and attention, which constitute key cognitive deficits of the disease (55). Although the studies with the highest methodological quality score (38, 39, 50) indicate a positive association of the anticholinergic load with cognitive deficiencies, the findings of one of the largest study of this systematic review (38) are characterized by a small effect size and are of dubious clinical importance. The various cognitive domains reported in the studies included may be due to the heterogeneity of the diagnostic evaluation tools used to assess cognitive functions.

Heretofore several systematic reviews have compared typical and atypical antipsychotic drugs in terms of their influence on the cognitive functions of patients with schizophrenia, with some studies suggesting that the use of atypical antipsychotics improves cognitive functions (56), whereas findings from other studies appear to support that both first and second generation antipsychotics have the same effect on cognitive functions (57). In general, cognitive impairment due to medication is claimed to be related to the affinity of drugs with receptors, and cognitive dysfunction is observed with drugs that are muscarinic cholinergic, a2 adrenergic, 5-HT2A/2C serotonergic, and D1/D2 dopaminergic blockers or agonists of 5-HT1A serotonin receptors. As far as the affinity of second-generation antipsychotics with anticholinergic receptors is concerned, this seems to vary (56). It is worth noting that the recent meta-analysis of Tani et al. (58), which included two randomized clinical trials that investigated antipsychotic dose reduction, found that 50% of SGAs dose reduction significantly improved neurocognitive performance. Thus, this indicates that atypical antipsychotics can also have harmful effects on neurocognitive function. These findings could also be attributed to the cumulative anticholinergic burden of antipsychotics as well as their antidopaminergic properties in the prefrontal cortex which have been associated with cognitive impairment (59). Therefore, in addition to the dose-dependent effects of antipsychotics on neurocognitive performance, the evaluation of antipsychotic therapy based on its anticholinergic profile as performed in our systematic review may provide a useful different approach for research and clinical purposes.

It is also worth discussing the findings of studies on antipsychotic clozapine (19, 51), which although considered a high potency anticholinergic agent, does not seem to affect cognitive performance to the same extent as other drugs with similar anticholinergic properties. These findings are in line with other studies which indicate the improvement of cognitive functions in patients with schizophrenia who were treated with antipsychotic clozapine (60, 61). The specificities regarding the action of clozapine are likely attributed to the distinct pharmacological characteristics related to its affinity with different receptors and thus the action on different neurotransmission systems (62). Furthermore, recent studies found that the ratio of clozapine to its active metabolite norclozapine (N-Desmethylclozapine) in plasma is associated with patients' cognitive performance. Clozapine has different pharmacodynamic properties and affinity to cholinergic, dopaminergic, and serotonergic receptors compared to its active metabolite and literature supports that lower clozapine/norclozapine ratios in clinically stable patients are associated with better cognitive outcome (63, 64). The metabolism of clozapine to norclozapine is induced by P450 enzymatic system and thus adjunctive treatments, caffeine consumption, smoking, and other factors such as age, race, and gender may alter this ratio between individuals (65, 66). Therefore, the consideration of all these confounding factors related to the complex pharmacological properties of clozapine is necessary for the interpretation of the findings of these studies.

In studies that assessed the impact of anticholinergic withdrawal on cognitive performance using different cognitive evaluation tools, successful discontinuation of anticholinergic medication by most patients was achieved, with a significant improvement in cognitive functions and without worsening of extrapyramidal symptoms or psychopathology compared to the baseline. Thus, this implies that anticholinergics do not cause long-term effects on cognition, which can even be reversed after withdrawal, and that long-term use may not be necessary in most patients receiving antipsychotics. Findings are also in agreement and support existing clinical guidelines, which do not recommend the prophylactic use of anticholinergic drugs, recommending anticholinergics co-administration only in the early stages of treatment if necessary and not for long-term use (67, 68). In the other hand, literature shows that despite the indications in clinical guidelines, the practice of administering anticholinergic therapy in combination with antipsychotics as well as antipsychotic polypharmacy varies across countries (69–73), with global rates of about 15% (74) and 20% (75) respectively. Furthermore, antipsychotic polypharmacy appears to be associated with cognitive impairment of schizophrenia patients, which could be due to the cumulative anticholinergic properties of drugs co-administered (74).

The systematic review underlines the urgent need for additional prospective studies (e.g., cohort studies, clinical trials) that will examine the longitudinal exposure in anticholinergic agents, with a more representative sample and longer monitoring duration to eliminate as many systemic errors and biases as possible and to draw more universal conclusions. More research is also certainly needed from a neurological point of view to explain and clarify the involvement of cholinergic neurotransmission and the general neurochemical mechanisms in the cognitive impairment of people with schizophrenia, since there are several hypotheses about various pathophysiological mechanisms that involve different neurochemical pathways and neurotransmitter systems including the role of muscarinic and nicotinic acetylcholine receptors (1, 76–78). Although several antipsychotics have been developed over the past decade to treat schizophrenia, cognitive rehabilitation drugs have not yet been approved by the FDA; therefore, cognitive deficits observed in the disease remain a huge scourge on the lives of millions of people around the world (79, 80). Not surprisingly, according to the findings of this systematic review, cholinesterase inhibitors have been proposed in several clinical studies as an additional therapy to standard antipsychotic treatments, to address cognitive decline in patients with schizophrenia. There are different cholinesterase inhibitor drugs with varying affinity either for acetyl cholinesterase (AChE) or butyryl cholinesterase (BChE) enzyme that act by blocking the cholinesterase enzyme from metabolizing ACh, leading to increased availability of ACh in neuron synapses (81). According to a previous systematic review, acetylcholinesterase inhibitor in combination with antipsychotic drug showed medium-sized improvements regarding the cognitive functions, particularly in the domains of attention, visual memory, verbal memory and language, and executive functioning (82). However, there is an urgent need for larger, well-designed randomized clinical trials for stronger evidence. Different approaches suggest in addition to memory enhancement drugs and cognitive training programs, which require more extensive study (83). Vinogradov et al. (53) reported a negative association between anticholinergic load and patients' response in the auditory training “based on neuroplasticity” programme, which has been supported by several studies as a promising approach to treating cognitive disorders in schizophrenia; therefore patients' medication history should be taken into account before assessing the effectiveness of the method (84, 85). Moreover, reassessment of the treatment already administered is important, since as we add to this review, it can affect cognitive functions, which may already be impaired due to the progressive degenerative nature of the disease.

As far as clinical practice is concerned, it is important for clinicians and other health professionals to assess the cumulative effects of anticholinergic drugs on cognition. Specifically, neurocognitive deficits can even explain about 20–60% of the variation in functional performance observed among patients with schizophrenia (24, 55, 86–88). A systematic review and meta-analysis of 50 studies highlights that the average percentage of patients with schizophrenia with clinical and social recovery characteristics was only 13.5% (89), thus demonstrating the need to allocate more resources to develop new research strategies for the treatment of this disease. Literature reports that the cognitive domains which have been negatively affected by anticholinergic load in most studies of this systematic review, directly affect the independence, social inclusion, and occupational activities of patients. Specifically, according to a study, global cognition is more closely related to the disability of the disease than individual neurocognitive domains. However, deficits in the domains of executive function and secondary verbal memory were associated with functional outcomes in community/daily activity (e.g., going to school, working). Short-term and secondary verbal memory were also largely associated with psychosocial skill acquisition. Furthermore, impairments in secondary verbal memory, vigilance, and to a lesser extent in executive function seemed to negatively affect social problem-solving skills. Composite scores show also a moderate to high association with the functional outcomes of the disease (86, 87). Hence, cognitive enhancement may have a significant impact on function, quality of life, patient well-being, as well as on the prevention of psychotic relapse (90). It is therefore recommended that the individual approach of each patient by healthcare professionals as well as the pharmacological treatment plan include the minimum necessary dose of antipsychotic medication, the restriction of polypharmacy, and caution in prescribing medicines with known anticholinergic activity. Finally, clinicians are required to reconsider the need for anticholinergic treatment before recommending any method or treatment for cognitive rehabilitation in schizophrenia.

### Quality Assessment and Risk of Bias

One of the main limitations of the studies included is the small sample size of the participants. Furthermore, amongst prospective cohort studies (48, 49), the lack of a control group makes the evaluation environment an important confounding factor and limits the interpretation of the results. Participant groups were matched by at least age and one other factor as well as in the studies without control group, a range of factors such as age, gender, education, and symptom severity were considered covariate adjustments in analysis. However, possible confounding factors such as the indirect treatment positive effect on cognition, medication adherence, and impact of other neurotransmitter systems have not been adjusted in most studies included. In addition, the inpatient setting in several studies could also affect the cognitive and daily function outcome and hasn't considered as a confounder factor (39, 40, 42, 44, 49, 50, 52). Regarding the included clinical studies of the systematic review (52, 53), they are characterized by low methodological quality with deficiencies mainly in terms of randomization and blinding methodology.

### Limitations

In terms of the present systematic review limitations, no meta-analysis could be performed due to the heterogeneity in the populations, methodology, and interventions of the included studies. Therefore, this affects the quality of the evidence presented because it is unclear whether the positive studies were favorable or whether there was a performance bias. Language bias is also possible as we could have missed non-English studies. Moreover, according to the hierarchy of evidence, most of the studies included on their methodological design are cross-sectional or retrospective studies with a limited number of participants. Hence, there is no time sequence between the exposure and the disease-outcome studied and cannot safely prove a causal relationship.

It is also worth mentioning that the studies included were based on tools which vary in terms of the classification of the anticholinergic burden of active substances, and which also have several limitations. Although the method of measuring SAA is the gold standard method for assessing anticholinergicity, this may reflect only a transitional cholinergic condition outside the brain, which confers an intuitive clinical capability but lacks a direct *in vivo* assessment of the central effect of anticholinergic medication. Furthermore, as a tool it cannot be used to draw conclusions about which medicine in particular may need to be discontinued to reduce the anticholinergic load, while the cost and availability of the method lead to the use of anticholinergic drug scales, which also have certain limitations (27, 41). In general, anticholinergic drug scales cannot calculate systemic drug exposure, brain delivery, and distribution of drugs or drug interactions that can often affect overall anticholinergic activity. Moreover, although the scales do not differ greatly in the classification of most medicines, discrepancies that could affect the outcome have been observed (e.g., in the case of quetiapine) (91, 92).

### Conclusion

The present systematic review shows that medication with increased anticholinergic load is possible to affect the cognitive functions of people with schizophrenia. However, based on different research methodologies and the clinical heterogeneity among various studies, it is not reasonable to make a definitive conclusion. Well-designed large prospective studies and randomized clinical trials are required to examine the effect of anticholinergic drug treatment on cognition in patients with schizophrenia. Based on these findings, clinicians are required to reconsider the need for anticholinergic treatment, with caution in prescribing medicines with known anticholinergic activity, before recommending any treatment for cognitive rehabilitation in schizophrenia.

## Data Availability Statement

The original contributions presented in the study are included in the article/supplementary material, further inquiries can be directed to the corresponding author/s.

## Author Contributions

RG carried out literature searches, appraised the articles, summarized the results, prepared the tables and figures, wrote the manuscript, and interpreted the results. DL interpreted the results. KG supervised the process, carried out literature searches, appraised the articles, summarized the results, wrote the manuscript, and interpreted the results. All authors contributed to the article and approved the submitted version.

## Conflict of Interest

The authors declare that the research was conducted in the absence of any commercial or financial relationships that could be construed as a potential conflict of interest.

## Publisher's Note

All claims expressed in this article are solely those of the authors and do not necessarily represent those of their affiliated organizations, or those of the publisher, the editors and the reviewers. Any product that may be evaluated in this article, or claim that may be made by its manufacturer, is not guaranteed or endorsed by the publisher.
